# Automated Segmentation of Coronary Arteries Based on Statistical Region Growing and Heuristic Decision Method

**DOI:** 10.1155/2016/3530251

**Published:** 2016-10-31

**Authors:** Yun Tian, Yutong Pan, Fuqing Duan, Shifeng Zhao, Qingjun Wang, Wei Wang

**Affiliations:** ^1^College of Information Science and Technology, Beijing Normal University, Beijing 100875, China; ^2^Department of Radiology, Navy General Hospital, Beijing 100048, China; ^3^Peking University Cancer Hospital & Institute, Key Laboratory of Carcinogenesis and Translational Research (Ministry of Education), Beijing 100142, China

## Abstract

The segmentation of coronary arteries is a vital process that helps cardiovascular radiologists detect and quantify stenosis. In this paper, we propose a fully automated coronary artery segmentation from cardiac data volume. The method is built on a statistics region growing together with a heuristic decision. First, the heart region is extracted using a multi-atlas-based approach. Second, the vessel structures are enhanced via a 3D multiscale line filter. Next, seed points are detected automatically through a threshold preprocessing and a subsequent morphological operation. Based on the set of detected seed points, a statistics-based region growing is applied. Finally, results are obtained by setting conservative parameters. A heuristic decision method is then used to obtain the desired result automatically because parameters in region growing vary in different patients, and the segmentation requires full automation. The experiments are carried out on a dataset that includes eight-patient multivendor cardiac computed tomography angiography (CTA) volume data. The DICE similarity index, mean distance, and Hausdorff distance metrics are employed to compare the proposed algorithm with two state-of-the-art methods. Experimental results indicate that the proposed algorithm is capable of performing complete, robust, and accurate extraction of coronary arteries.

## 1. Introduction

Over the past decades, coronary artery disease (CAD) has been the main cause of human deaths in the world [1]. Many factors can lead to CAD, and, of these, stenosis caused by atherosclerosis is the most common. Coronary arteries are usually extracted first to diagnose stenosis. An inaccurate segmentation of coronary arteries can result in fatal false treatments because a missing segment or mixed extraction of other structures can lead to the oversight of existing stenosis or improper narrow lumen detections.

Many studies have been conducted on automated or semiautomated segmentation of coronary arteries on computed tomography angiography (CTA) images. Automated segmentation methods can automatically segment regions of interest of images, without any human intervention. However, the complexity of such methods is usually relatively high [2, 3]. Compared with automated segmentation methods, semiautomated segmentation methods [4, 5] require the interactions of therapists, making the methods less convenient than automated methods. However, the performances of semiautomated methods are sometimes better than those of the automated methods. Öksüz et al. [4] proposed a hybrid method composed of threshold preprocessing, vessel enhancement, and traditional 3D region growing. Threshold preprocessing in this method retains many uninterested regions. In addition, traditional 3D region growing requires human interactions and lacks accuracy. Thus, the precision and automation of this method must be improved.

Kitamura et al. [5] proposed a novel coronary segmentation method based on multilabel graph cuts; this method utilizes higher-order potentials to impose shape priors. However, the method derives a limitation from the Hessian-based features, which cannot distinguish a very wide variety of structures. In addition, the method requires human interactions. Research on automated segmentation methods has been making great progress recently, and automated methods are gradually becoming popular in clinical diagnosis. However, automated methods have problems in both efficiency and accuracy. To solve this problem, Lugauer et al. [6] proposed a lumen segmentation method for coronary CTA. They utilized a Markov random field formulation with convex priors that rely on the training of a large dataset. Their method is sensitive to the dataset and its training and also proved to be inefficient because the dataset analysis is time-consuming. Zheng et al. [7] proposed a machine learning-based method that can gain better performance than empirically designed measurement (e.g., the widely used Hessian-based method), but the learning process requires the analysis of a large expert-annotated dataset, which is also very time-consuming. Zhou et al. [8] presented a method following the steps of heart region extraction, multiscale coronary artery response method for vascular structure enhancement, automated detection of seed points, and 3D dynamic balloon-tracking method for coronary arteries tracking. However, the EM algorithm used in the heart region extraction has low efficiency because of the huge amount of points in the CTA volume. Meanwhile, Bouraoui et al. [9] introduced an automated method based on advanced mathematical morphology techniques. They employed a blurry grey-level hit-or-miss transform method to detect seed points automatically. However, the 13 structure candidates employed in their method occasionally fail to detect seed points because they cannot cover all patient conditions. Many of the works mentioned above use region growing as a part of the segmentation process, and these region growing methods are not fully automated and lack robustness and accuracy. In addition, the precision of coronary artery extraction needs to be improved.

In this paper, we present a novel scheme for extracting coronary arteries from CTA images. First, the heart region is extracted using a multi-atlas-based approach. Second, the vessel structures are enhanced via a 3D multiscale line filter. Next, a set of seed points is detected automatically through threshold preprocessing and a subsequent morphological operation. Based on the set of seed points, statistics-based region growing is applied. Upon generating the results by setting conservative parameters, a heuristic decision method is used to obtain the desired result. Each step of the algorithm is fully automated for an efficient and automated pipeline. The proposed algorithm outperforms two state-of-the-art methods, which are used for comparison. The experiments are carried out on eight-patient multicenter multivendor cardiac CTA volume data, and the DICE similarity index, mean distance, and Hausdorff distance metrics are employed to compare the different methods.

The rest of the paper is organized as follows. We introduce two related works in Section 2 to be compared with our proposed method.  Section 3 describes the proposed method in detail. We validate the proposed method in Section 4 through the well-known evaluation methodology mentioned above. Conclusions are provided in Section 5.

## 2. Related Works

### 2.1. Öksüz et al

The algorithm proposed by Öksüz et al. [4] consists of five stages, of which three are related to coronary artery segmentation. First, pulmonary vessels are removed by performing the thresholding and morphological procedures. Then, Frangi vesselness filter [10] is applied on the processed data. Finally, vessel segmentation is achieved by successively performing 3D region growing and fast marching.

The Frangi vesselness filter used in their algorithm is based on the eigenvalues of the Hessian matrix:

(1) $$\begin{array}{c} H=\left[\begin{array}{ccc} I_{xx} & I_{xy} & I_{xz}\\ I_{yx} & I_{yy} & I_{yz}\\ I_{zx} & I_{zy} & I_{zz} \end{array}\right], \end{array}$$

where *I*_*xx*_, *I*_*zy*_, and so forth are the partial second derivatives of image *I*(*x*, *y*, *z*). An ideal bright 3D line is defined as

(2) $$\begin{array}{c} I\left(\;x,y,z\;\right)=\exp\left(\;\frac{-\left(x^{2}+y^{2}\right)}{2\sigma^{2}}\;\right), \end{array}$$

where the direction of the maximum second derivative is identical to the direction of the *z*-axis (i.e., the direction of the line), and its value is zero. Any second derivative orthogonal to the *z*-axis has a negative value at each point in the central region of the line cross section. Ideally, the conditions of a bright line can be regarded as  *λ*_1_≅0, *λ*_2_≅*λ*_3_ ≪ 0.

In the method of Öksüz et al., the pulmonary vessels may not be completely removed by simply setting the thresholding and morphological operations. Furthermore, the region growing in their method is not fully automated.

### 2.2. Zhou et al

The algorithm proposed by Zhou et al. [8] consists of four stages. First, the heart region is extracted using the EM algorithm. Then, a 3D multiscale filter is applied to the heart region. Next, seed points are detected automatically on the processed data. Finally, a dynamic balloon-tracking algorithm is applied to track the coronary arteries.

The idea of this method is similar with our algorithm. However, the EM algorithm employed in the heart extraction is time-consuming, which is unacceptable in the clinical diagnosis process. Moreover, multi-atlas-based heart extraction is more precise than the EM algorithm. The multi-atlas-based method is based on the registration of the standard images to obtain the optimal parameters, which determine the deformation of the reference images to form the extracted goal. Although this method requires also a large amount of computation, the Gaussian pyramid model can be employed to improve the computational efficiency. The dynamic balloon tracking can also terminate in the coronary segments where high-grade stenosis exists. In comparison, statistical region growing, based on a group of seed points, can handle this situation. As a result, our method performs better in the diseased coronary segments.

## 3. Proposed Methodology

The proposed method, which can robustly and automatically extract complete coronary arteries, has four steps as follows: (1) heart segmentation, (2) vessel enhancement, (3) extraction of seed points, and (4) coronary artery extraction.

### 3.1. Segmentation of the Heart Region

We apply a two-stage registration method denoted as an optimization problem searching for an optimal transformation *T*, which minimizes the dissimilarity between a fixed image *I*_*F*_(*x*)  and a moving image *I*_*M*_(*x*):

(3) $$\begin{array}{c} \hat{T}=\arg\min C\left(\;I_{F}\left(\;x\;\right),I_{M}\left(\;T\left(x\right)\;\right)\;\right). \end{array}$$

In the equation above, *C* is a cost function that measures the dissimilarity between two images.

In the first stage, an affine registration is used to spatially align the fixed and moving images roughly. The cost function is defined as

(4) $$\begin{array}{c} C\left(\;C,I_{F},I_{M}\;\right)=\frac{1}{\left|\sigma_{F}\right|}\underset{x_{i}\in \sigma_{F}}{\sum }{\left[\;I_{F}\left(\;x_{i}\;\right)-I_{M}\left(\;T_{C}\left(\;x_{i}\;\right)\;\right)\;\right]}^{2}, \end{array}$$

where *σ*_*F*_ is the fixed image domain. This equation is calculated using image samples that are randomly chosen in each iteration in the entire image domain. Then, 256 iterations of the gradient descent optimizer are performed.

In the second stage, a B-spline registration is utilized. The result of the affine registration is used as the initialization of B-spline registration. In each iteration, one voxel is selected randomly in the entire image domain. The remaining image samples are picked in a 50 mm square neighborhood around that voxel. The algorithm optimizes the localized similarity measure using the equation

(5) LC,IF,IM=∑m∈LM ∑f∈LFpf,m;C·log2⁡pf,m;CpFfpMm;C,

where *L*_*F*_ and *L*_*M*_ are sets of regularly spaced intensity bin centers,  *p* is the discrete joint probability, and *p*_*F*_ and *p*_*M*_ are the marginal discrete probabilities of the fixed and moving images obtained by summing *p* over *f* and *m*, respectively.

Furthermore, a Gaussian pyramid model is applied during the registration to improve the computational efficiency. The transformation parameters calculated in (1) are propagated to label the regions that must be segmented to the patient images. Finally, majority voting is used to combine the labeled atlas images and generate the final heart segmentation results. The atlas images employed in our method are similar to those used in [11].

### 3.2. Vessel Structures Enhancements

After the heart region is extracted, a 3D multiscale line filter, previously described by Sato et al. [12], is applied to segment the curvilinear structures in the heart region. The method used here is similar to the Frangi vesselness filter, which has been introduced in Section 2.1 (i.e., the method proposed by Öksüz et al.).

### 3.3. Automated Seed Points Detection

As coronary arteries are enhanced in CTA images, a threshold operator plays an important role in noncardiovascular removal. The threshold value used in this process should be conservative in consideration of the various pathologies of different patients. Thus, in this study, a threshold of 120 HU is used in our method, and a 3D erode filter is employed to suppress noise points. The kernel of the erosion process should be sufficiently large to ensure that the remaining points belong to coronary arteries. In our method, the erosion kernel is defined as a 4 × 4 × 3 cube. Therefore, a point set is attached to the coronary arteries, wherein all of the points are to be applied in the next step.

### 3.4. Coronary Artery Extraction

By using all the previously detected seed points, coronary arteries are extracted using a statistics-based growing method, which identifies voxels with similar statistics via connectivity. The method is based on the iterative computation of the statistical information of voxel intensities included in the current region. As for each seed point, the mean and variance across a 26-connected neighborhood are calculated to define a range:

(6) $$\begin{array}{c} I=\left[\;m-v*d,\;m+v*d\;\right], \end{array}$$

where *I* represents the consistent interval; *m* and *d* stand for the mean and standard deviation of the seed point, respectively; and *v* is a bounds control parameter, which is automatically set through the heuristic decision manner. 26-neighborhood voxels with intensities within this range are included in the region. If the seed points were included in the growing region, they are removed from the seed point set. Thus, the growing efficiency can be improved. It is noteworthy that some parts of coronary arteries may not be included by the region growing of a seed point, but they may be included by the region growing of other seed points. Therefore, using a seed set to carry out the statistics-based region growing, not only can the robustness of the proposed algorithm be enhanced, but also the integrity of the segmented coronary arteries can be improved.

After this initial segmentation is calculated, the mean and variance, of all voxels from the current segmented coronary arteries, are computed again to define a new intensity range. The intensity range is used to detect whether intensities of the voxels in the neighborhood of the current segmentation fall within the range. If a voxel's intensity is within the interval, the voxel is included in the segmented arteries. This process is repeated until no new voxels are added or a specified number of iterations are reached. In this study, the number of iterations was set to 5.

**Figure 1:**
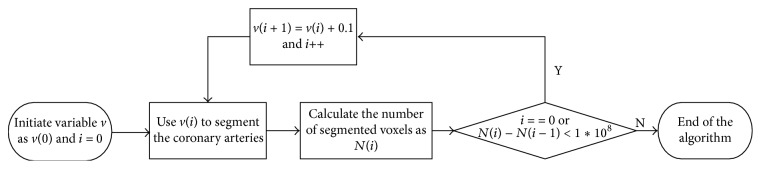
Flow chart of the statistics-based method.

Whenever a seed point is provided, the mean and standard deviation can be calculated automatically. However, *v* cannot be automatically obtained. Hence, a heuristic decision method is presented to acquire *v* without human interactions, and its flow chart is shown in Figure 1. The process is described below.

(1) Initialize *v*_0_ to 1.0 and make *i* (set to 0 at first) refer to the number of times the method should be repeated.
(2) Perform segmentation using *v*_*i*_ and calculate *N*_*i*_ (i.e., the number of segmented points). If *N*_*i*_ is calculated for the first time, go to step (4); else continue to step (3).
(3) Compare *N*_*i*_ with *N*_*i*−1_. If the difference between them is over 1*∗*10^8^, the algorithm is ended.
(4) Gradually increase *v*_*i*_ by a step of 0.1; go to step (2).

**Figure 2:**
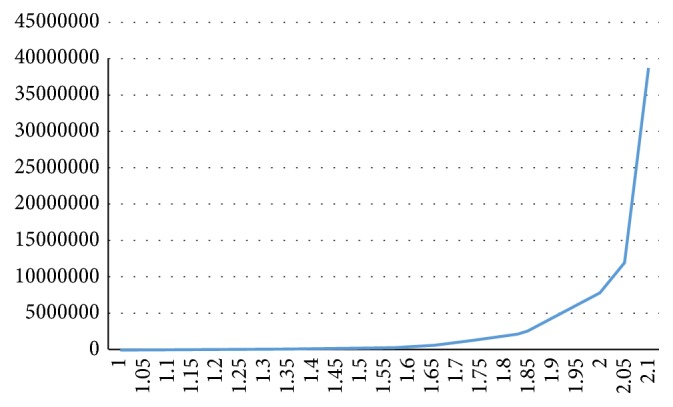
Sketch map of the mutation between the desired and redundant results (the horizontal axis represents the incremental variable  *v*, and the vertical axis represents the number of the segmented voxels).

This heuristic decision method is based on the fact that a mutation between the desirable and redundant segmentations exists, as shown in Figure 2. Specifically, before the advent of oversegmentation, the segmented results of each increment of *v*, compared with that of the previous *v*, the change of the number of the segmented voxels is less than 1 × 10^7^ orders of magnitude. If the change of the number of the segmented voxels is greater than 1 × 10^8^, this means “mutation.” The reason is that the redundant segmentation almost comprises the whole heart, whereas the coronary arteries are relatively small parts of the heart region.

## 4. Result and Discussion

### 4.1. Data

CTA provides a visualization of the whole chest, including vessel lumen, atherosclerotic, and stenosis, without the invasive catheterization procedure [13]. Thus, CTA is less harmful compared with the traditional 2D X-ray angiography because only the contrast medium is required to be injected before proceeding to CTA. Meanwhile, its 3D reconstruction capability is highly suitable for the treatment of CAD.

We employ CTA images as materials in the testing and development of our method. The detailed acquisition parameters of CTA are shown in Table 1.

### 4.2. Result and Validation

**Figure 3:**
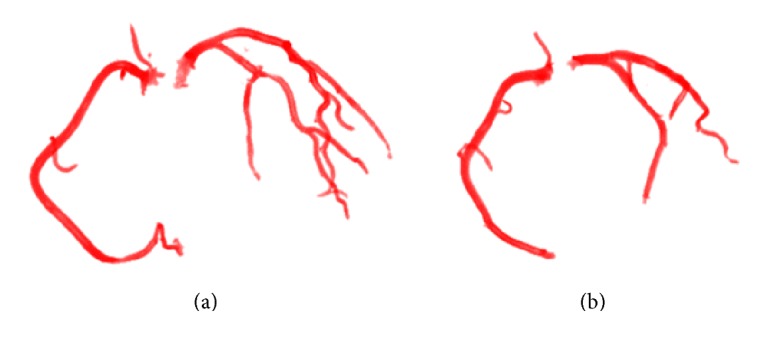
Extraction results by our method from different patient volume CTA data. (a) A patient who suffers from low-grade stenosis in both the right coronary artery (RCA) and the left coronary artery (LCA). (b) A patient who suffers from high-grade stenosis in the LCA and low-grade stenosis in the RCA.

The validation process of the proposed method was built based on the publicly accessible standardized coronary artery evaluation framework presented by Kirişli et al. [14]. The method presented in this paper was implemented using C++ within the VolView open-source platform (http://www.kitware.com/opensource/volview.html).  Figure 3 shows images of the left and right coronary arteries extracted by our method from two-patient volume CTA data.

In the experiment, two state-of-the-art methods (those presented by Öksüz et al. [4] and Zhou et al. [8]) were compared to our method using the volumetric overlap (DICE) and max/mean surface distance (MAXSD/MSD) as metrics. DICE is the dice similarity coefficient, which is used to evaluate the volumetric cardinality of different algorithms.  Table 2 lists the DICE, MAXSD, and MSD values calculated separately for healthy and diseased arteries over a dataset of eight patients with different grades of stenosis (D and H denote diseased and healthy vessels, resp.). As can be seen, the proposed method performs best on both healthy and diseased vessel segments for DICE and MAXSD metrics. Furthermore, the method achieved the highest rank compared to the two state-of-the-art methods.

**Figure 4:**
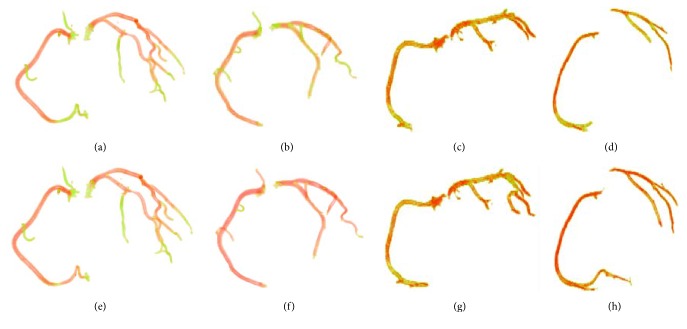
Comparison of our method with Öksüz's method [4] and Zhou's method [8] using four patients with different stenosis. Rows 1 and 2 show the comparisons between our method and Öksüz's method [4] and Zhou's method [8], respectively. Each dataset result from the different comparisons is shown in every column.

Visual results and segmentation comparisons between the proposed method and one of the two state-of-the-art methods are shown in Figure 4. The first row of Figure 4 shows the comparison between our method and that proposed by Öksüz et al. [4] using four patients with different stenosis in both the LCA and RCA. The second row of Figure 4 shows the comparison between our method and that proposed by Zhou et al. [8] using the same dataset as that of the first row. Each dataset result from the different comparisons is shown in every column. The red vessels in Figure 4 are common segments extracted by both the proposed and state-of-the-art methods, and the green vessels are the segments extracted by the proposed method and missed by the traditional methods. It can be seen from the first row of Figure 4 that Öksüz's method is only capable of extracting the thick vessels, which is not connective. The reason is that Öksüz's method employs the thresholding and morphological operations to extract arteries based on the globe information of volume data, which fails to cope with the coronary arteries with different intensities. It can be seen from the second row of Figure 4 that Zhou's method obtains a better result than that of Öksüz's method and is capable of extracting most coronary arteries. However, some weak arteries are still missing, as shown in the first column of the second row in Figure 4. The reason is that the artery segmentation is terminated in advance by the dynamic tracking balloon where there is a high-grade stenosis. It is clearly seen from Figure 4 that our method is capable of accurately extracting the thick and thin arteries, and they are red and green. At the same time, the segmented coronary arteries appear with better connectivity. The excellent performance of our method is attributed to the automated seed points detection and the elaborately exploited statistics-based region growing.

**Figure 5:**
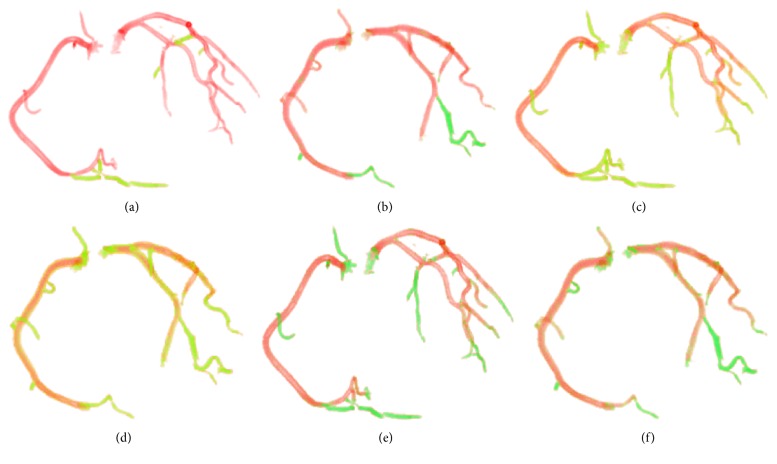
Comparison results between each method and the gold standard. (a) Our method and the gold standard using a sample patient with low-grade stenosis. (b) Our method and the gold standard using a sample patient with high-grade stenosis. (c) The method proposed by Öksüz et al. [4] and the gold standard using a sample patient with low-grade stenosis. (d) The method proposed by Öksüz et al. [4] and the gold standard using a sample patient with low-grade stenosis. (e) The method proposed by Zhou et al. [8] and the gold standard using a sample patient with low-grade stenosis. (f) The method proposed by Zhou et al. [8] and the gold standard using a sample patient with high-grade stenosis.

Figure 5 shows the difference between the segmentation methods and gold standards derived from the manual segmentation of expert radiologists in Beijing's Navy General Hospital to ensure the validity and authenticity of our experiment. In Figure 5 the common parts are painted red, and the differences are painted green. Two different patients with low- and high-grade stenosis were employed as samples in the comparison of each method with the gold standard.  Figure 5(a) shows our method and the gold standard using a sample patient with low-grade stenosis.  Figure 5(b) shows our method and the gold standard using a sample patient with high-grade of stenosis.  Figure 5(c) shows the method proposed by Öksüz et al. [4] and the gold standard using a sample patient with low-grade stenosis.  Figure 5(d) shows the method proposed by Öksüz et al. [4] and the gold standard using a sample patient with low-grade stenosis.  Figure 5(e) shows the method proposed by Zhou et al. [8] and the gold standard using a sample patient with low-grade stenosis.  Figure 5(f) shows the method proposed by Zhou et al. [8] and the gold standard using a sample patient with high-grade stenosis. As can be seen from Figure 5, our method yielded a robust segmentation for each volume data. This can be attributed to the fact that a segment missing by one seed point in the proposed method can be supplemented by other seed points in the subsequent growing process because of the employment of a novel seed point detection method, and the heuristic decision that determines the desired results automatically ensures the absence of human interaction in the pipeline. The detailed information on the equipment used in our experiment is as follows: Intel® Core™ i7-3770 CPU, 8.0 GB RAM, 64-bit Ubuntu 14.04. The time consumed by the proposed method was less than 60 seconds for each set of volume data.

## 5. Conclusion

We proposed a fully automated segmentation of coronary arteries from 3D cardiac CTA (CCTA). This algorithm is a combination of various technologies, including multi-atlas, multiscale vascular enhancement, morphological, and statistical decision technologies. First, the heart region is extracted by a multi-atlas-based approach, which is capable of segmenting the heart due to the employed prior knowledge from the reference heart atlas set. At the same time, the registration efficiency is improved by a Gaussian pyramid model employed. Then, a 3D multiscale vessel filter is applied to enhance the coronary artery structures, which effectively enhances the coronary artery contrast, because the shape information of the blood vessel is considered. Subsequently, seed points are detected automatically through threshold preprocessing and a morphological operation. Based on the set of seed points, statistics-based region growing is applied, which grows the coronary arteries in virtue of the local statistical information of the seek point. Thus, the connectivity of the arteries can be well guaranteed. Finally, results are obtained by setting conservative parameters, and then a heuristic decision is employed to automatically obtain the desired result. The coronary arteries are gradually segmented from the CCTA data in a coarse-to-fine manner. No manual interaction is involved in the entire segmentation process because each required parameter is searched using novel specific algorithms. Hence, the proposed algorithm is capable of performing complete, robust, and accurate extraction of coronary arteries.

## Figures and Tables

**Table 1:** CT acquisition parameters.

Parameter name	Value
Voxel spacing	0.33 × 0.33 × 0.4 mm^3^
Resolution	512 × 512 voxels/slice
Slice thickness	0.8 mm
Tube voltage	120 kV
Exposure time	1833 ms
Series description	75%
Table height	89 mm

**Table 2:** Comparison of the segmentation results (D and H denote diseased and healthy vessels, resp.).

Method	DICE D [%]	DICE H [%]	MSD D [mm]	MSD H [mm]	MAXSD D [mm]	MAXSD H [mm]	Rank avg.
Proposed method	**0.71**	**0.76**	0.34	0.41	**2.47**	**2.75**	**4.2**
Öksüz et al. [4]	0.60	0.68	0.45	0.55	3.94	6.48	6.9
Zhou et al. [8]	0.69	0.72	**0.32**	**0.39**	2.87	3.20	4.4
